# Evaluation of bone allograft processing methods: Impact on decellularization efficacy, biocompatibility and mesenchymal stem cell functionality

**DOI:** 10.1371/journal.pone.0218404

**Published:** 2019-06-20

**Authors:** Alexander Rasch, Hendrik Naujokat, Fanlu Wang, Andreas Seekamp, Sabine Fuchs, Tim Klüter

**Affiliations:** 1 Experimental Trauma Surgery, Department of Trauma and Orthopedic Surgery, University Medical Center Schleswig-Holstein, Campus Kiel, Kiel, Germany; 2 Department of Oral and Maxillofacial Surgery, University Medical Center Schleswig-Holstein, Campus Kiel, Kiel, Germany; Università degli Studi della Campania, ITALY

## Abstract

In an ever-aging society the demand for bone-defect filling grafts continues to gain in importance. While autologous grafting still prevails as the gold standard, allografts and xenografts present viable alternatives with promising results. Physiochemical properties of a graft strongly depend on the processing method such as the decellularization protocol. In addition, the physiochemical characteristics are critical factors for a successful integration of the graft after the implantation and might influence mesenchymal stem cell function in therapeutic approaches combining grafts and autologous mesenchymal stem cells (MSCs). Several decellularization methods have been proposed, however it still remains unclear which method results in favorable physiochemical properties or might be preferred in stem cell applications. In the first part of this study we compared two decellularization approaches resulting in chemically processed allografts (CPAs) or sonication-based processed allografts (SPAs). Each decellularization approach was compared for its decellularization efficacy and its influence on the grafts’ surface texture and composition. In the second part of this study biocompatibility of grafts was assessed by testing the effect of extraction medium on MSC viability and comparing them to commercially available allografts and xenografts. Additionally, grafts’ performance in terms of MSC functionality was assessed by reseeding with MSCs pre-differentiated in osteogenic medium and determining cell adhesion, proliferation, as well as alkaline phosphatase (ALP) activity and the degree of mineralization. In summary, results indicate a more effective decellularization for the SPA approach in comparison to the CPA approach. Even though SPA extracts induced a decrease in MSC viability, MSC performance after reseeding was comparable to commercially available grafts based on DNA quantification, alkaline phosphatase activity and quantification of mineralization. Commercial Tutoplast allografts showed overall the best effects on MSC functionality as indicated by extraction biocompatibility testing as well as by comparing proliferation and osteogenic differentiation.

## Introduction

According to the 3^rd^ edition report of The Burden of Musculoskeletal Diseases in the United States the number of people who received care after suffering bone fractures raised to more than 23 million [1]. As fracture incidence has been shown to have a bimodal distribution with a high fracture rate for elderly people [2], an increase in fracture prevalence in an ever-aging society can be expected in the future. While most fractures heal within 3–8 weeks [3] about 5–10% of fractures end in non-unions [4], which together with infected non-unions, high-energy injuries and bone loss due to i.e. tumor resection cause critical bone defects [5]. The treatment of bone defects is challenging for the attending surgeon and in many cases bone grafts are required. While autologous bone grafting is currently still considered the gold standard [6, 7] some limitations are associated with autologous grafting, such as risk of infections, additional surgical sites and limited bone supply [8]. Allografts and xenografts present viable alternatives to autografts as they solve the problem of limited autologous bone supply and do not require an additional surgical site for graft harvesting [9]. However, allogenic and xenogenic grafting can carry the risk of infection [10] and may induce an immunological reaction in the graft recipient [11]. Thus, a successful usage of allografts and xenografts *in vivo* requires a thorough removal of immune response eliciting material, such as bone marrow content or potential pathogens [12]. This is usually achieved by decellularizing the bone graft using a combination of chemical substances (Triton X-100, sodium dodecyl sulfate, hydrogen peroxide), enzymes (DNAse, trypsin) and physical treatment (centrifugation, sonication, temperature treatment). While decellularization may be viewed as the central step in graft processing, donor selection [13] and graft harvesting [14] have also shown to exert an influence on the graft’s properties. Properties of the bone graft such as surface area, surface structure, chemical composition and mechanical stability may be altered by the processing of the graft [15–17] and may influence the implantation *in vivo* or the growth of bone forming cells such as MSCs, for instance. Hence, special attention has to be paid in terms of processing.

Mesenchymal stem cells (MSCs) [18–20] have been studied in depth also in conjunction with evaluating bone grafts. MSCs exhibit a multipotent differentiation capacity but are lacking pluripotent characteristics as i.e. human dental follicle stem cells [21, 22]. The osteogenic potential of MSCs and the option to isolate them from the bone marrow make them ideally suited for bone tissue engineering applications. Bone grafts have shown the potential to support differentiation of MSCs into the osteogenic lineage [23, 24], which is also regulated at an epigenetic level [25]. Furthermore, the combined application of MSCs and bone grafts demonstrated enhanced healing properties of large bone defects *in vivo* after implantation [26, 27]. In addition to their ability to produce new bone tissue the therapeutic potential of MSCs has in part been attributed to paracrine effects that MSCs exert via cytokines and growth factors [28] on adjacent MSCs and bone tissue upon integration *in vivo* [29]. At the same time, cytokine secretion by MSCs and bone forming cells can be influenced by the implant’s properties as it has been shown for CaP containing grafts being able to influence the cells’ secretome towards an osteogenic profile through adenosine signaling [30]. Physiochemical properties of the graft have a direct influence on cell adherence and cell proliferation of MSCs after seeding onto bone grafts [31–33] and hence play an important role upon application of seeded grafts *in vivo*. Ideally, the processing of bone grafts for tissue engineering applications should decellularize the graft completely, inactivate any potential harmful pathogens, maintain biomechanical stability and in conjunction with stem cell applications demonstrate osteoconductive or osteoinductive properties combined with high biocompatibility as defined by Williams [34].

Several decellularization methods, such as decellularization based on chemical treatment [35], sonication [36] and irradiation [37] have been proposed, yet it remains unclear which method results in favorable properties for *in vivo* use as well as favorable reseeding properties in conjunction with MSCs. In this study we compare two decellularization methods adapted from published protocols for bone grafts based on chemical treatment [35] or sonication [36]. The effect of each method on the graft’s surface texture, composition and decellularization including bone barrow removal was investigated. Decellularized grafts as well as two commercially available grafts, one allograft (Tutoplast) and one xenograft (Bio-Oss), were further subjected to element analysis and MSC viability assays with extracts derived from the grafts. Commercially available grafts were included in this study as additional references for standardized graft processing. Self-decellularized grafts as well as commercially available grafts were reseeded with MSCs pre-differentiated in osteogenic medium and cell adhesion, proliferation and osteogenic activity was assessed in order to compare their performance in conjunction with MSCs.

## Materials and methods

### Ethical approval

The use of human tissue and cells was approved by the local ethical advisory board of the Medical Faculty of Christian-Albrechts-Universität in Kiel, Germany including the consent from the individual donors.

### Allografts decellularized by chemical treatment

Femoral heads were obtained from patients undergoing total hip replacement surgery. Donors consisted of five female and five male donors, ranging from 42 to 93 years (mean 73.2 years, SD ± 18.9 years) and 51 to 80 years (mean 69.8 years, SD ± 14.5 years), respectively. Femoral heads were cut into discs with a thickness of 5 mm from which cylinders with a diameter of 6 mm were cut using a hollow drill. Prior to decellularization bone cylinders were stored in -80°C without the addition of buffers or liquids.

After defrosting chemical decellularization was started by 3 freeze/thaw cycles with liquid nitrogen. This was followed by incubating bone grafts two times for 24 hours, each in 750 μL 2% (v/v) Triton X-100 (Sigma-Aldrich, Taufkirchen, Germany) diluted with phosphate-buffered saline (PBS) (Fisher Scientific, Loughborough, UK). All solutions were sterile filtered before use. After treatment with Triton X-100, bone grafts were incubated for 24 hours at room temperature in 750 μL 1% (w/v) sodium dodecyl sulfate (SDS) (Sigma-Aldrich, Darmstadt, Germany) solution diluted in PBS. Then, they were washed with PBS for 30 min on an orbital shaker and incubated with 200 U/mL DNAse I (Sigma-Aldrich, Darmstadt, Germany) solution at 37°C for 12 hours. The procedure was finished by washing the allografts three times for 2 hours with PBS on an orbital shaker. Allografts decellularized by this protocol are henceforth referred to as chemically processed allografts (CPAs).

### Allografts decellularized by Sonication

Grafts decellularized using sonication are referred to as sonication-based processed allografts (SPAs). Bone grafts were defrosted from -80°C, submerged in 1 mL of distilled water preheated to 60°C and sonicated for 15 min at 20 kHz with an amplitude of 12 microns (Mk2 sonicator, MSE, London, UK). Allografts were then rinsed with PBS until solution became clear and were then placed in 750 μL PBS on an orbital shaker for 2 hours at 60°C. This was succeeded by a wash-centrifuge sequence repeated three times. The sequence consisted in a washing step with distilled water at 60°C on an orbital shaker and a centrifugation step at 1850 x g for 15 min at room temperature. The first washing step was performed for 30 min while the second and third for 10 min. Allografts were then sonicated first in 1 mL 3% (v/v) hydrogen peroxide (Sigma-Aldrich, Darmstadt, Germany) solution diluted with PBS at 60°C and then in 1 mL 70% (v/v) ethanol (Th. Geyer, Renningen, Germany) diluted with distilled water at room temperature, both for 10 min at 20 kHz and with an amplitude of 12 microns. Allografts were then placed in distilled water at 60°C for 10 min on an orbital shaker and centrifuged for 15 min at 1850 x g at room temperature. Decellularization was finished by placing allografts in distilled water at room temperature for 30 min on an orbital shaker. After completion of either CPA or SPA protocol, allografts were thoroughly washed and stored without the addition of buffers or solutions in 4°C until use.

### Commercial allografts and xenografts

Tutoplast (RTI Surgical, Alachua, FL, USA) and Bio-Oss (Geistlich, Wolhusen, Switzerland) grafts were acquired as cancellous bone blocks from bovine and human origin, respectively, and were cut into cylinders with the same dimensions as used for decellularization. They are referred to as Bio-Oss processed xenografts (BPXs) and Tutoplast processed allografts (TPAs).

### Histological examination of decellularized allografts

Histological examination was performed by fixation of bone cylinders in 4% paraformaldehyde (PFA) (Morphisto, Frankfurt am Main, Germany) for 24 hours. After samples were embedded in polymethyl methacrylate (PMMA) they were polished, cut and mounted onto slides. After slide thickness was adjusted to 40–60 μm, staining was performed by consecutively incubating slides for 2 min with 0.1% (v/v) formic acid (Merck, Darmstadt, Germany), 90 min in 20% (v/v) methanol (Merck, Darmstadt, Germany) and 2 min in toluidine blue staining solution (Merck, Darmstadt, Germany). Solutions were diluted in distilled water and in-between each step slides were washed also in distilled water. Images were taken with EVOS FL Auto 2 Imaging System (ThermoFisher Scientific, Bothell USA).

### Scanning electron microscopy

Specimens were prepared for scanning electron microscopy (SEM) by fixation in 3% (v/v) glutaraldehyde (Sigma-Aldrich, Darmstadt, Germany) diluted in PBS. Following fixation for 24 hours, samples were treated with ethanol gradients ranging from 50% up to 99% (v/v) ethanol. Prior to imaging 3 μL of hexamethyldisilazane (ThermoFisher, Kandel, Germany) were applied and samples were gold sputtered with a 10 nm thick layer. Images were taken with Philips XL 30 CP SEM (Philips, Amsterdam, Netherlands).

### DNA quantification of decellularized allografts

In order to assess the degree of decellularization DNA content was quantified from CPAs, SPAs, TPAs and BPXs. Additionally, controls that had not been treated other than storing in -80°C were analyzed as a reference. Grafts were placed in 2 mL Eppendorf tubes and 1 mL nuclease-free water (Ambion, Carlsbad, CA, USA) was added. Then, three freeze/thaw cycles in -80°C and sonication for 30 seconds at 20 kHz and with an amplitude of 12 microns was performed. After centrifuging at 2000 x g for 5 min at room temperature the supernatant was transferred, and the total DNA amount quantified with Quant-iT PicoGreen dsDNA assay kit (Molecular probes, Eugene, OR, USA). DNA quantification for each bone graft material (CPAs, SPAs, TPAs and BPXs) was performed for two cylinders from 3 donors in technical triplicates. The DNA amount was quantified by fluorescence with a microplate reader (TECAN, Maennedorf, Switzerland) at an excitation wavelength of 485 nm and an emission wavelength of 535 nm.

### Energy-dispersive X-ray spectroscopy

Energy-dispersive x-ray spectroscopy (EDX) analysis was performed using a Philips XL 30 CP SEM. Prior to analysis samples were sputtered with carbon. The SEM was operated with 25 kV and examined areas on grafts were chosen so that 2100 counts per second (CPS) were registered and dead time was 30–35%. Measurements were performed for a period of 200 live seconds (Lsec). Three donors were used for each bone graft material. Each cylinder was measured twice at two different surface areas of the graft.

### Isolation and culture of MSCs

MSCs were isolated from cancellous bone of femoral heads obtained from patients undergoing total hip replacement surgery. Isolation was performed as mentioned previously [38, 39]. In brief, cancellous bone fragments were washed thoroughly with PBS and PBS wash solution centrifuged at 400 x g for 5 min. The cell pallet was then collected in Dulbecco’s Medium Essential Medium (DMEM)/Ham’s F-12 medium (Biochrom, Berlin, Germany) supplemented with 20% (v/v) fetal bovine serum (FBS) (Sigma, Taufkirchen, Germany) and 1% (v/v) Penicillin/Streptomycin (Biochrom, Berlin, Germany) and seeded at a density of 2x10^6^ cells/cm^2^ in T175 flasks coated with collagen type I (Corning, Bedford, MA, USA). After splitting cells culturing was continued using 10% (v/v) FBS. In passage 2 osteogenic differentiation of MSCs was induced by incubation in osteogenic differentiation medium (ODM) for a minimum of two weeks consisting of DMEM/Ham’s F-12, 10% (v/v) FBS, 1% (v/v) Pen/Strep, 10 mM β-glycerophosphate (Sigma-Aldrich, Darmstadt, Germany), 0.1 μM dexamethasone (Sigma-Aldrich, Darmstadt, Germany) and 50 μM ascorbic acid (Sigma-Aldrich, Darmstadt, Germany). Medium was changed three times a week. Experiments with MSCs were conducted with cells in passage no. 4 and 5.

### Biocompatibility testing using extracts obtained from decellularized bone grafts

In order to assess the biocompatibility of decellularized grafts, extracts of CPAs, SPAs, TPAs and BPXs were prepared according to ISO 10993. Then, the effect of extracts on MSC viability was assessed using MTS (3-(4,5-dimethylthiazol-2-yl)-5-(3-carboxymethoxyphenyl)-2-(4-sulfophenyl)-2H-tetrazolium). Extracts were created by incubating decellularized bone grafts in 750 μL ODM at 37°C for either 24 hours or 72 hours and stored at -20°C until use. MSCs were seeded at a density of 45,500 cells/cm^2^ in a 96 well-plate (Sarstedt, Nümbrecht, Germany) that had been pre-coated with 33.6 μg/mL collagen type I solution diluted in PBS. After allowing cells to attach for 24 hours ODM was aspirated, extracts were defrosted, centrifuged at 12,000 x g for 5 min, added to the cells and incubated for 48 hours. Metabolic activity of MSCs was assessed using CellTiter 96 AQuerous One Solution Cell Proliferation Assay (Promega, Madison, WI, USA) according to manufacturer’s instructions. Optical density was measured with a microplate reader (TECAN, Maennedorf, Switzerland) at 490 nm. Three MSC donors and three distinct bone donors for CPAs and SPAs were used to assess biocompatibility in technical triplicates. Data were depicted in relation to untreated controls.

### Seeding of MSCs onto constructs

After coating grafts with 10 μg/mL fibronectin (Millipore, Temecula, CA, USA) 200,000 MSCs were drop seeded in a volume of 100 μL ODM onto precoated CPAs, SPAs, TPAs and BPXs. After cells were left to attach to grafts for 1 hour at 37°C 650 μL of ODM was added and medium was exchanged three times per week.

### Evaluation of MSC-seeded constructs by CLSM and SEM microscopy

After 7 days of cultivation MSC-seeded constructs were fixed in 4% PFA solution followed by three wash cycles in PBS for 15 min and twice for 5 min. Samples were then treated with 0.5% (v/v) Triton X-100 diluted in PBS for 20 min. This was followed by washing in PBS. Intracellular F-actin was stained with 5 μg/mL tetramethylrhodamine (TRITC)-conjugated phalloidin (Sigma-Aldrich, Darmstadt, Germany) in 1% (v/v) bovine serum albumin (BSA) (millipore, Kankakee, USA) in PBS for 30 min. Hoechst 33258 (Sigma-Aldrich, Darmstadt, Germany) at a concentration of 2 μg/mL in PBS for 15 min was used for nuclear counterstain. Samples were imaged by confocal laser scanning microscope (CLSM) (LSM 510 Meta, Zeiss, Oberkochen, Germany). After CLSM MSC-seeded constructs were prepared for SEM as described above.

### DNA quantification of MSC-seeded constructs

In order to evaluate cell attachment and cell proliferation of MSCs on SPAs, TPAs and BPXs, DNA quantification was performed on day 1, day 7 and day 14 after cell seeding. MSC-seeded constructs were transferred into new 48 well-plate wells and DNA was extracted by performing three freeze/thaw cycles at -80°C after the addition of nuclease-free water, followed by sonication at a frequency of 20 kHz and an amplitude of 12 microns for 30 seconds. At each time point DNA quantification was performed for two independent grafts and technical replicates for 3 donors. DNA quantification was performed as described above.

### ALP assay

ALP activity was measured from supernatants of MSC-seeded constructs after 1, 7 and 14 days using an alkaline phosphatase assay kit (Abcam, Cambridge, UK). Measurements were run in technical triplicates and ALP activity measurements were performed according to manufacturer’s instruction. Measurements were normalized to 1 μg DNA at the indicated time points. At each time point ALP quantification was performed for two independent grafts and technical replicates for 3 donors.

### Alizarin red S quantification

Alizarin Red S staining was performed to assess mineralization of MSCs on SPAs, TPAs and BPXs. After 7 and 14 days of cultivation MSC-seeded constructs were fixed in 4% PFA for 1 hour, washed thrice with PBS and consecutively incubated in 500 μL Alizarin Red S staining solution (40 mM, Merck, Darmstadt, Germany) for 60 min at room temperature on an orbital shaker. After washing MSC-seeded constructs with distilled water Alizarin Red S was extracted using a 10% (w/v) cetylpyridinium chloride (CPC) solution (Carl Roth, Karlsruhe, Germany) for 48 hours. Finally, Alizarin Red S was quantified by measuring the optical density at 560 nm and calculated in accordance with the standard curve. At each time point Alizarin Red S quantification was performed for two independent grafts and technical replicates for 3 donors.

### Statistical analysis

Statistical analysis was carried out using GraphPad Prism 7. Statistical significance was assessed using ANOVA as indicated in the individual experiments. A *p*-value of *p* < 0.05 (* *p* < 0.05, ** *p* < 0.01, *** *p* < 0.001, **** *p* < 0.0001) was considered to be statistically significant.

## Results

### Histological examination of decellularized allografts

**Fig 1:**
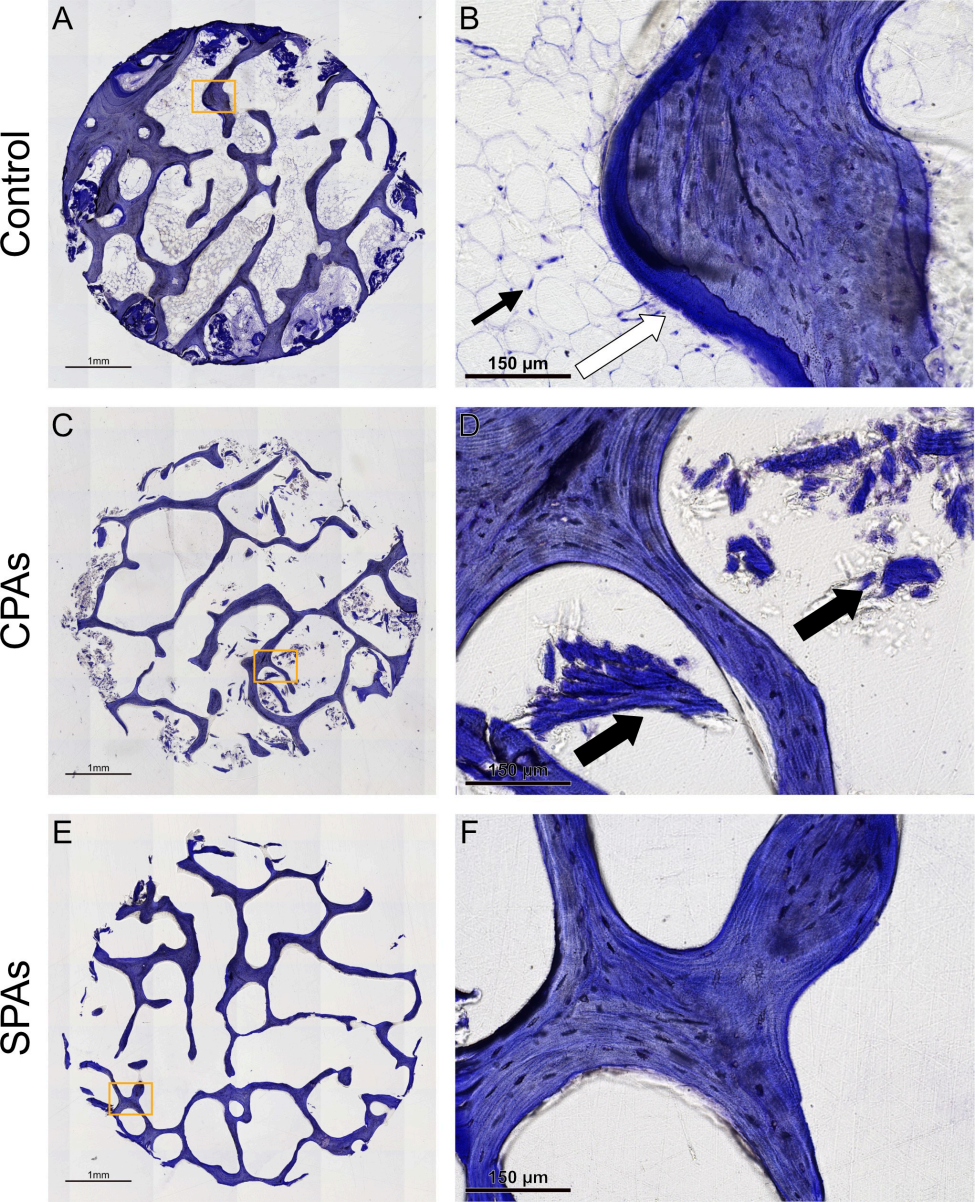
Histological assessment of CPAs, SPAs and controls. Toluidine blue stained sections show untreated control allografts (A, B), CPAs (C, D) and SPAs (E, F). Images document less cells and residues of bone marrow in SPAs compared to CPAs and control. White arrow (B) points to deposited matrix, thin black arrow (B) to peripherally located nuclei of adipocytes and black thick arrows (D) point to trabecular fragments. Images A, C, E show stitched images using 42 individual images. Scale bar: A, C, E = 1 mm, B, D, F = 150 μm.

Histological examination of decellularized bone grafts with toluidine blue staining was performed in order to assess the decellularization efficacy of chemical and sonication-based decellularization protocols (Fig 1). Untreated controls (Fig 1A) show trabecular structures in blue and marrow cavities extensively filled with bone marrow. Higher magnification of this sample (Fig 1B) shows deposited matrix peripherally to the trabecular structures (white arrow). Furthermore, cells showing the morphology of adipocytes can be detected in the marrow cavity (thin black arrow). CPAs contain only little to no marrow content (Fig 1C). However, some trabecular fragments in the marrow cavities (thick black arrow) can be seen (Fig 1D). SPAs show completely empty marrow cavities and very distinct trabecular structures (Fig 1E). Higher magnification (Fig 1F) confirms marrow cavities void of any material.

### SEM images of decellularized allografts

**Fig 2:**
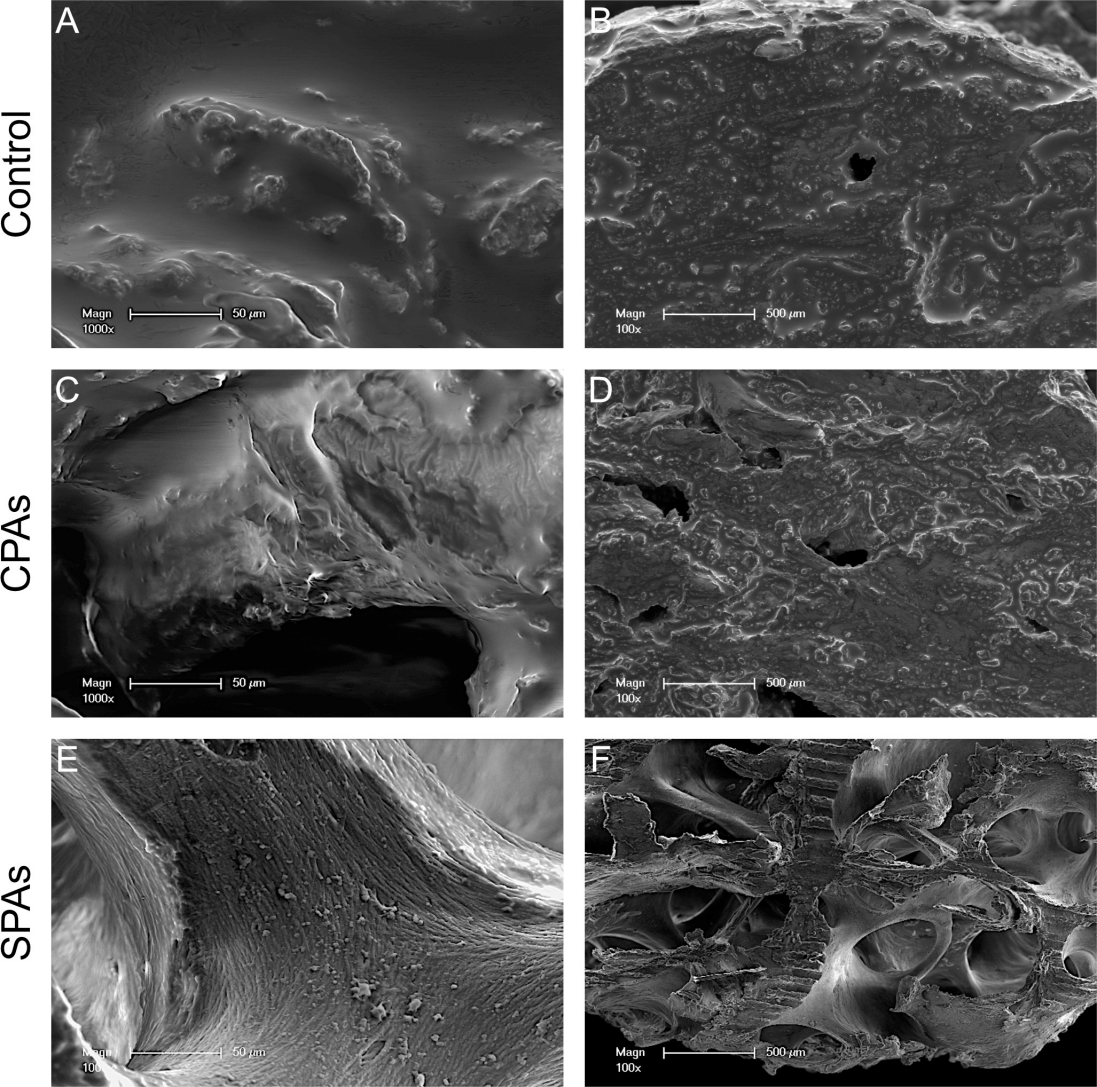
SEM assessment of CPAs, SPAs and controls. SEM images show untreated control allografts (A, B), CPAs (C, D) and SPAs (E, F). SEM images depict more empty marrow cavities for SPAs in comparison to CPAs and control. Scale bar: A, C, E = 50 μm, B, D, F = 500 μm.

In order to confirm histological examination and to further assess the surface topography of decellularized bone grafts SEM images were taken (Fig 2). SEM images of untreated controls (Fig 2A) display a similar surface structure compared to CPAs (Fig 2C). Surface structure of SPAs (Fig 2E) differs visually from controls and CPAs. Additionally, SPAs (Fig 2F) display more trabecular structures devoid of bone marrow or soft tissue in the cavities compared to CPAs (Fig 2D). CPAs show a surface topography that resembles those of untreated control grafts (Fig 2B).

### DNA quantification of decellularized allografts

**Fig 3:**
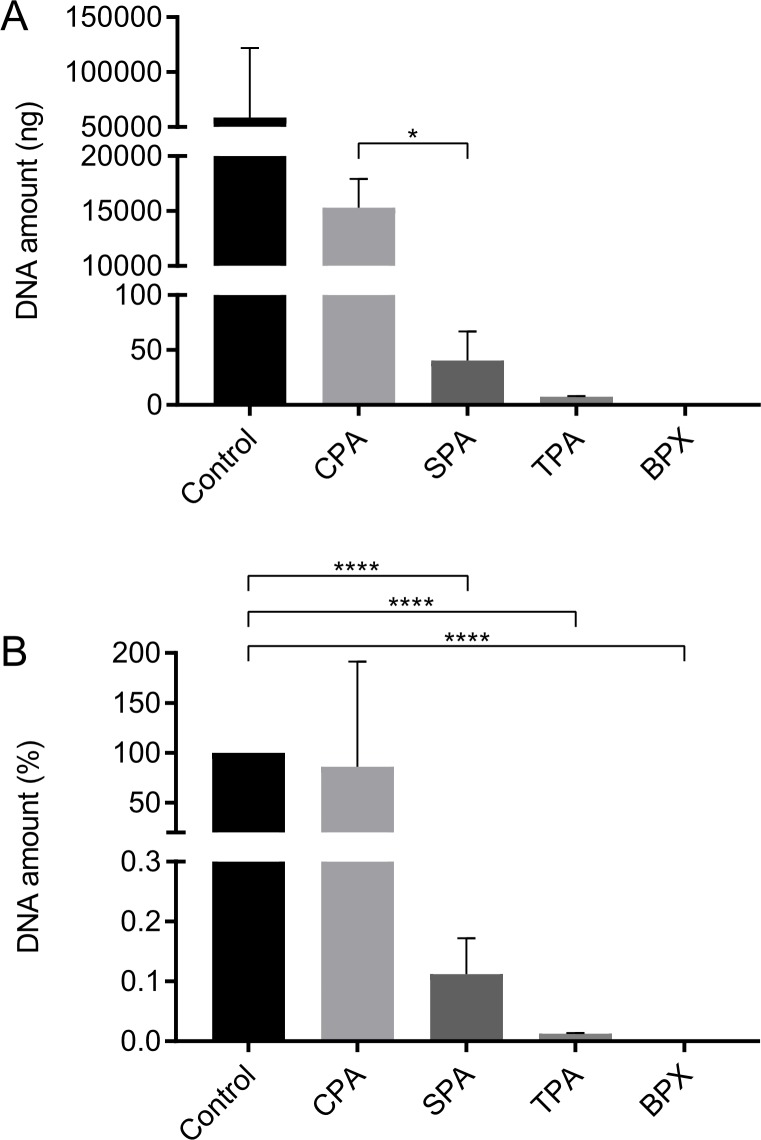
DNA quantification of CPAs, SPAs, and commercially available allografts (TPAs) and xenografts (BPXs). Highest amounts of residual DNA were detected in CPAs which differed significantly from SPAs, based on Tukey’s multiple comparison in conjunction with ANOVA. TPAs and BPXs show low amounts of DNA (A). Values of SPAs, TPAs and BPXs are significantly lower compared to control (B), as assed by Tukey’s multiple comparison in conjunction with ANOVA (*n* = 3 donors, two independent grafts were measured per donor, * *p* < 0.05, **** *p* < 0.0001).

In order to further verify and quantitively assess the efficacy of the decellularization protocols for CPAs and SPAs, DNA was quantified. Further, as additional references and controls DNA contents of commercially available and standardized grafts (TPAs and BPXs) were measured (Fig 3). DNA is depicted as absolute values (Fig 3A) and in relation to control allografts that had not been decellularized (Fig 3B). Decellularized CPAs revealed a mean value of 15,304 ng DNA. SPAs yielded a mean value of 40.3 ng while controls showed an average of 58,279 ng. TPAs and BPXs yielded a total amount of 7.4 ng and 0.49 ng DNA, respectively. In relation to untreated controls, CPAs showed a non-significant DNA reduction to 85.98%, while SPAs showed a significant DNA reduction to 0.11%. Compared to controls, DNA levels of TPAs and BPXs showed a significant DNA reduction to 0.01% and 0%, respectively. These data suggest a much more effective decellularization for the SPA compared to CPA processed grafts. Decellularization efficacy for SPA was tentatively lower but still comparable to the two commercially available grafts used as additional references.

### EDX analysis

**Fig 4:**
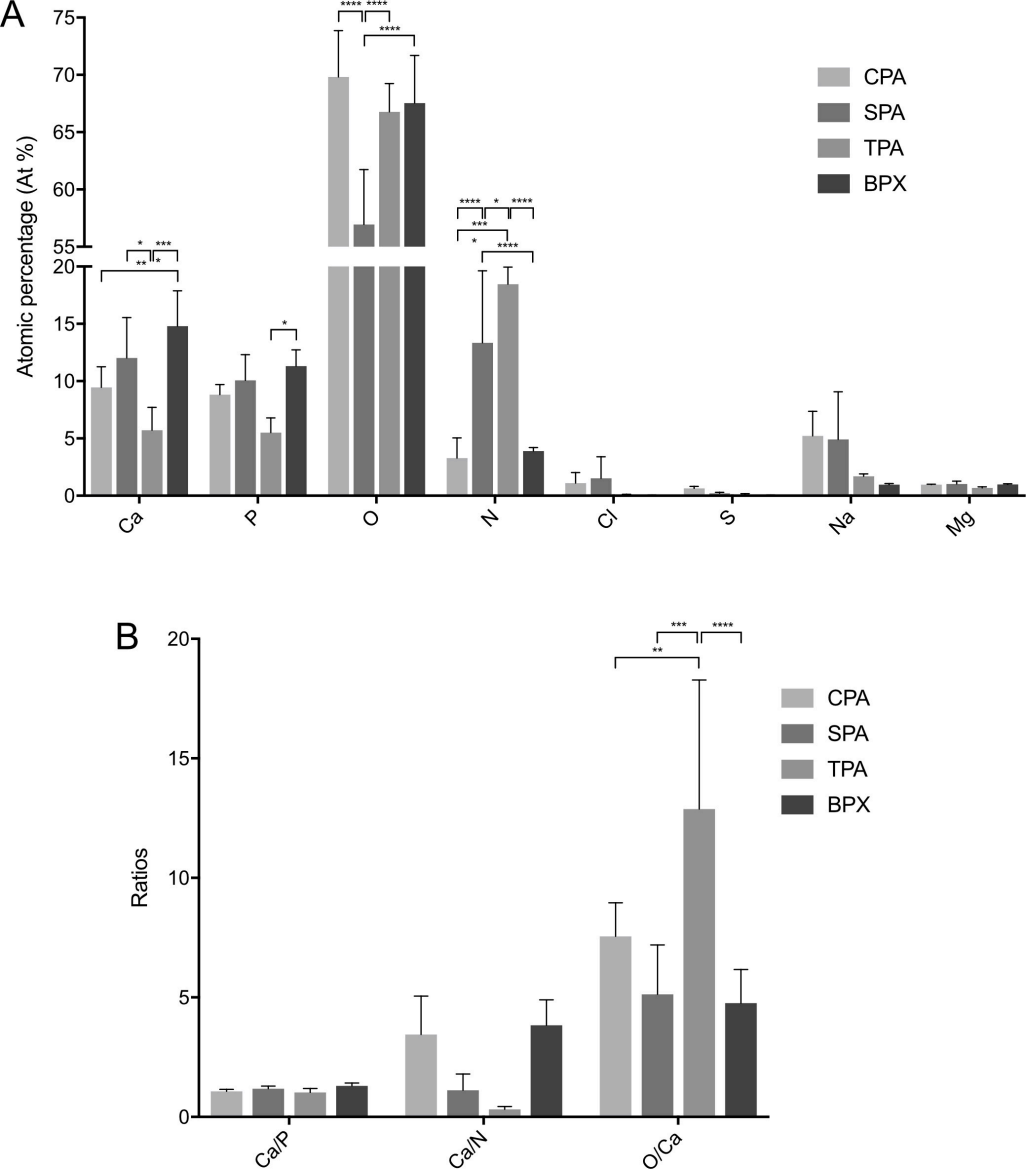
EDX analysis of CPAs, SPAs, TPAs and BPXs. Analysis shows element composition in atomic percentage (At%) (A) and Ca/P, Ca/N and O/Ca ratios (B) in order to assess chemical composition. TPAs show the highest amount of N, a low Ca/N ratio and high O/Ca ratio while BPXs show the highest values for Ca and P. Statistics are based on Tukey’s multiple comparison in conjunction with ANOVA (*n* = 3 donors, two different surface areas were measured per donor, * *p* < 0.05, ** *p* < 0.01, *** *p* < 0.001, **** *p* < 0.0001).

Element analysis was performed by EDX spectroscopy to reveal the elemental composition of CPAs and SPAs and compare it to commercially available TPAs and BPXs. Fig 4A shows significant differences in atomic percentage (At%) between CPAs and SPAs for oxygen (O) (12.88%) and nitrogen (N) (-10.06%). Among all bone grafts TPAs displayed the least amount of calcium (Ca) and phosphorous (P) and the highest amount of N, differing significantly from all other tested grafts. BPXs showed the highest values in Ca and P, including significant differences to CPAs and TPAs with regard to Ca and significant differences to TPAs with regard to P. Values for N in BPXs were low, showing significant differences to SPAs and TPAs. Since it previously has been shown that grafts with a Ca/P ratio of ∼ 1.43 can induce osteogenesis [30], Ca/P ratios were displayed (Fig 4B). In addition, Ca/N and O/Ca ratios were displayed as N and O are ubiquitous in many organic compounds. For the bone essential Ca/P ratios did not differ significantly between grafts. However, TPAs showed the lowest Ca/N ratio whereas the O/Ca ratio was highest, showing significant differences compared to all other grafts.

### Biocompatibility testing using extracts obtained from decellularized bone grafts

**Fig 5:**
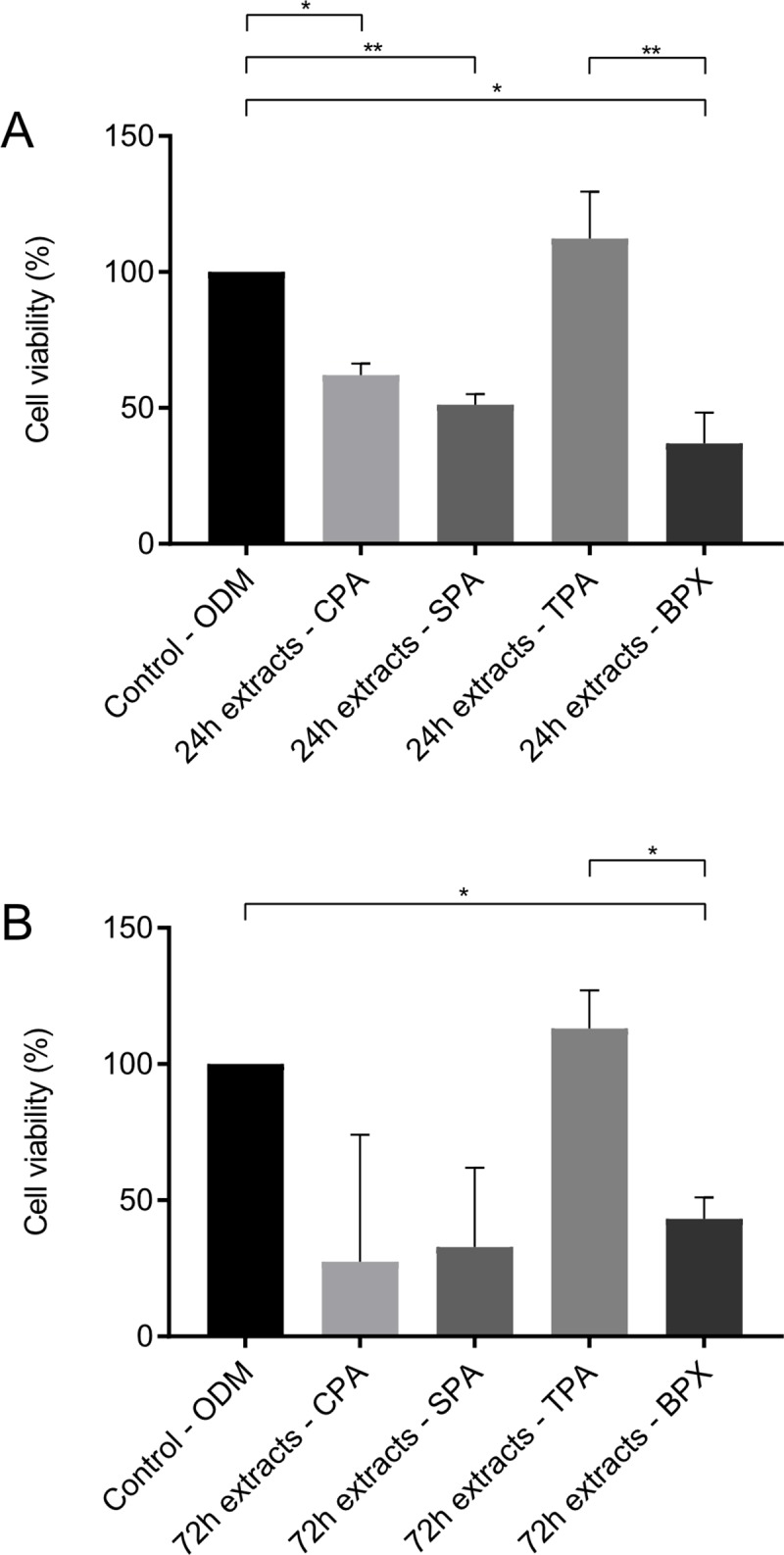
Biocompatibility testing performed for CPAs, SPAs, TPAs and BPXs with MSCs. Allografts and xenografts were incubated in ODM for 24 hours (A) or 72 hours (B) to create extracts. Extracts were then added to MSCs on 96 well-plates after cells attached to wells for 24 hours. After 48 hours of exposure to extracts MTS assay was performed. In contrast to all other grafts TPAs show no reduction in cell viability. Statistics are based on Tukey’s multiple comparison in conjunction with ANOVA (*n* = 3 graft donors, extracts were added to 3 different MSC donors, * *p* < 0.05, ** *p* < 0.01).

In order to assess potentially harmful substances leaking out of the processed bone grafts, extracts were created by adding ODM to CPAs, SPAs, TPAs and BPXs and biocompatibility was assessed by MTS assay using MSCs. Extracts were created by incubating allografts and xenografts in ODM for either 24 hours or 72 hours and were then added to MSCs on 96 well-plates 24 hours after seeding. After 48 hours of exposure to extracts MTS assay was performed. Results of MTS assay (Fig 5) using 24-hour extracts showed a significant decrease in cell viability of CPA extracts (62.04%), SPA extracts (51.17%) and BPX extracts (36.94%) (Fig 5A). TPA extracts showed a non-significant increase (112.28%). Extracts gained by 72-hour extraction time showed similar results (Fig 5B). Accordingly, the highest values in cell viability were observed for TPAs.

### Evaluation of MSC-seeded constructs by CLSM and SEM microscopy

**Fig 6:**
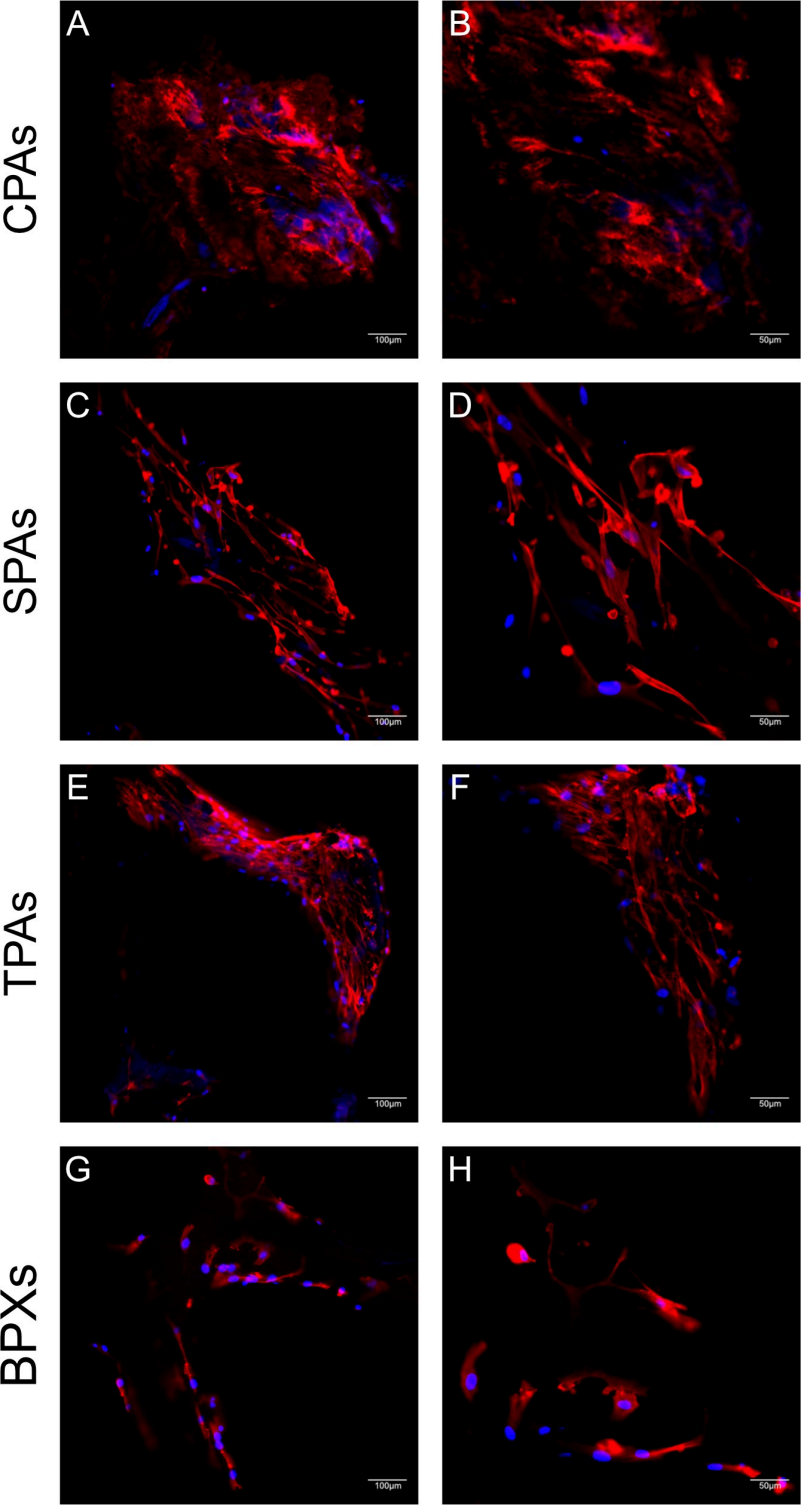
Morphology assessment of seeded MSCs on CPAs, SPAs, TPAs and BPXs using CLSM. Images display MSCs 7 days after seeding onto CPAs (A, B), SPAs (C, D), TPAs (E, F) and BPXs (G, H) stained with TRITC-conjugated phalloidin / Hoechst nuclear stain co-stain. Images of CPAs depict cells but as well high levels of background fluorescence. MSCs on BPXs show a lower cell density than cells on SPAs and TPAs. Scale bars: A, C, E, G = 100 μm, B, D, F, H = 50 μm.

While previous results characterized SPAs and CPAs after the decellularization, the following results were obtained after these grafts were reseeded with MSCs. First, CLSM images of cell-seeded constructs were taken 7 days after seeding (Fig 6). F-Actin staining was used to visualize the cytoskeleton and cells were depicted in combination with nuclear counterstain. Images of cell-seeded CPAs (Fig 6A and 6B) display some background fluorescence in the red channel due to the material properties interfering to some extend with a distinct fluorescence pattern for the cytoskeleton. Nevertheless, several nuclei are visible, confirming the presence of cells on CPAs. Images of cell-seeded SPAs (Fig 6C) show abundant numbers of MSCs revealing an elongated morphology with centrally located nuclei (Fig 6D). CLSM images of cell-seeded TPAs (Fig 6E) show a high cell density. Upon higher magnification (Fig 6F) cells seem to display a smaller morphology than cells on SPAs (Fig 6D). MSCs on BPXs (Fig 6G and 6H) show cellular protrusions with a morphology not as elongated compared to SPAs and TPAs. Additionally, the cell density appears to be lower than on MSC-seeded SPAs and TPAs.

**Fig 7:**
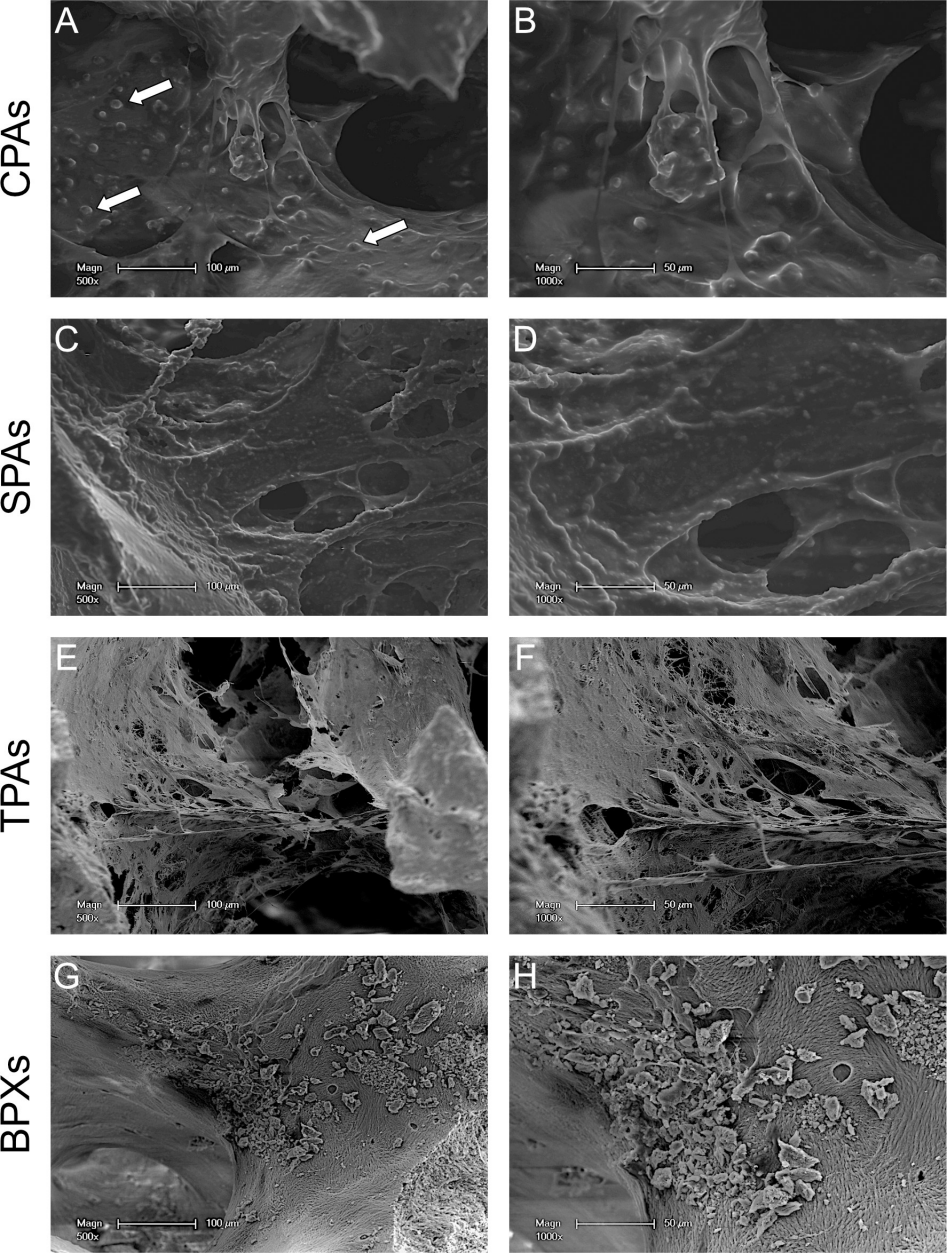
Morphology assessment of seeded MSCs on CPAs, SPAs, TPAs and BPXs using SEM. Images show MSCs 7 days after seeding onto CPAs (A, B), SPAs (C, D), TPAs (E, F) and BPXs (G, H). CPAs, SPAs and TPAs show elongated spindle-like cells while MSCs on BPXs appear to be fewer in number. White arrows point to nuclear protrusions. Scale bars: A, C, E, G = 100 μm, B, D, F, H = 50 μm.

SEM images of MSC-seeded constructs shown in Fig 7 reflect findings from CLSM images. Cell-seeded CPAs (Fig 7A and 7B) display elongated cells and nuclear protrusions (white arrows). MSC-seeded SPAs also show elongated cells with several nuclear protrusions (Fig 7C and 7D). Congruent to CLSM images MSCs on TPAs (Fig 7E) display a smaller morphology with a high number of cellular processes (Fig 7F). Seeded BPXs (Fig 7G and 7H) show few cells with few elongated processes analogous to Fig 6H.

### DNA quantification of MSC-seeded constructs

**Fig 8:**
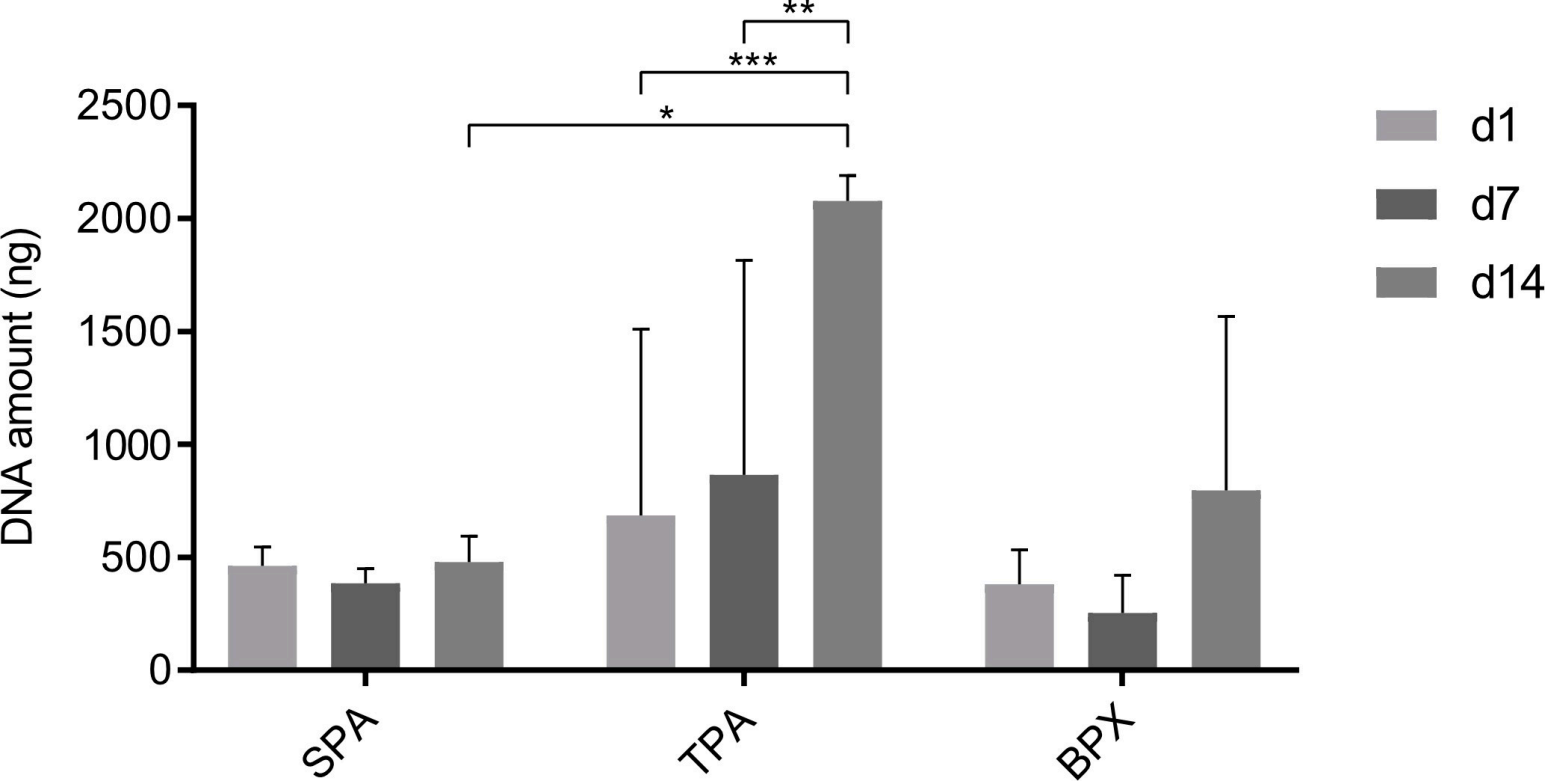
DNA quantification of MSCs 1, 7 and 14 days after seeding onto SPAs, TPAs and BPXs. TPAs show a significant increase in DNA from day 1 and day 7 to day 14, suggesting proliferation of MSCs. Further, DNA levels differed significantly between SPAs and TPAs on day 14 indicating better cell growth on TPAs. DNA background values determined for empty grafts (compare Fig 3) were subtracted from values obtained after MSC-seeding. Statistics are based on Tukey’s multiple comparison in conjunction with ANOVA (*n* = 3 MSC donors and 3 graft donors, two independent MSC-seeded constructs were quantified per donor, * *p* < 0.05, ** *p* < 0.01, *** *p* < 0.001).

Cell adhesion to grafts (day 1) as well as the rate of proliferation (day 7 and 14) of MSCs on SPAs, TPAs and BPXs was determined by DNA quantification (Fig 8). The DNA content of corresponding unseeded grafts was subtracted as background for each group. In consequence to the results from Fig 3 where CPAs did not reveal a sufficient level of decellularization, CPA data were excluded from quantitative evaluation of the MSC performance after reseeding.

DNA quantification after reseeding showed a significant increase of MSC numbers grown on TPAs on day 14 (2077 ng) compared to day 1 (685.7 ng) and day 7 (864.5 ng), thus indicating the proliferation of MSCs on TPAs in the investigated time frame. In addition, after 14 days significant differences in DNA amounts were observed for TPAs and SPAs, suggesting a better performance of MSCs on TPAs after reseeding. Although the cell growth on BPXs also showed a tentative increase up to 14 days (796.5 ng), DNA quantification data did not reveal significant differences for the investigated time points. Similar amounts of DNA were observed on day 1 for TPAs, SPAs and BPXs suggesting that the initial adhesion is comparable.

### Osteogenic activity determined by ALP assay

**Fig 9:**
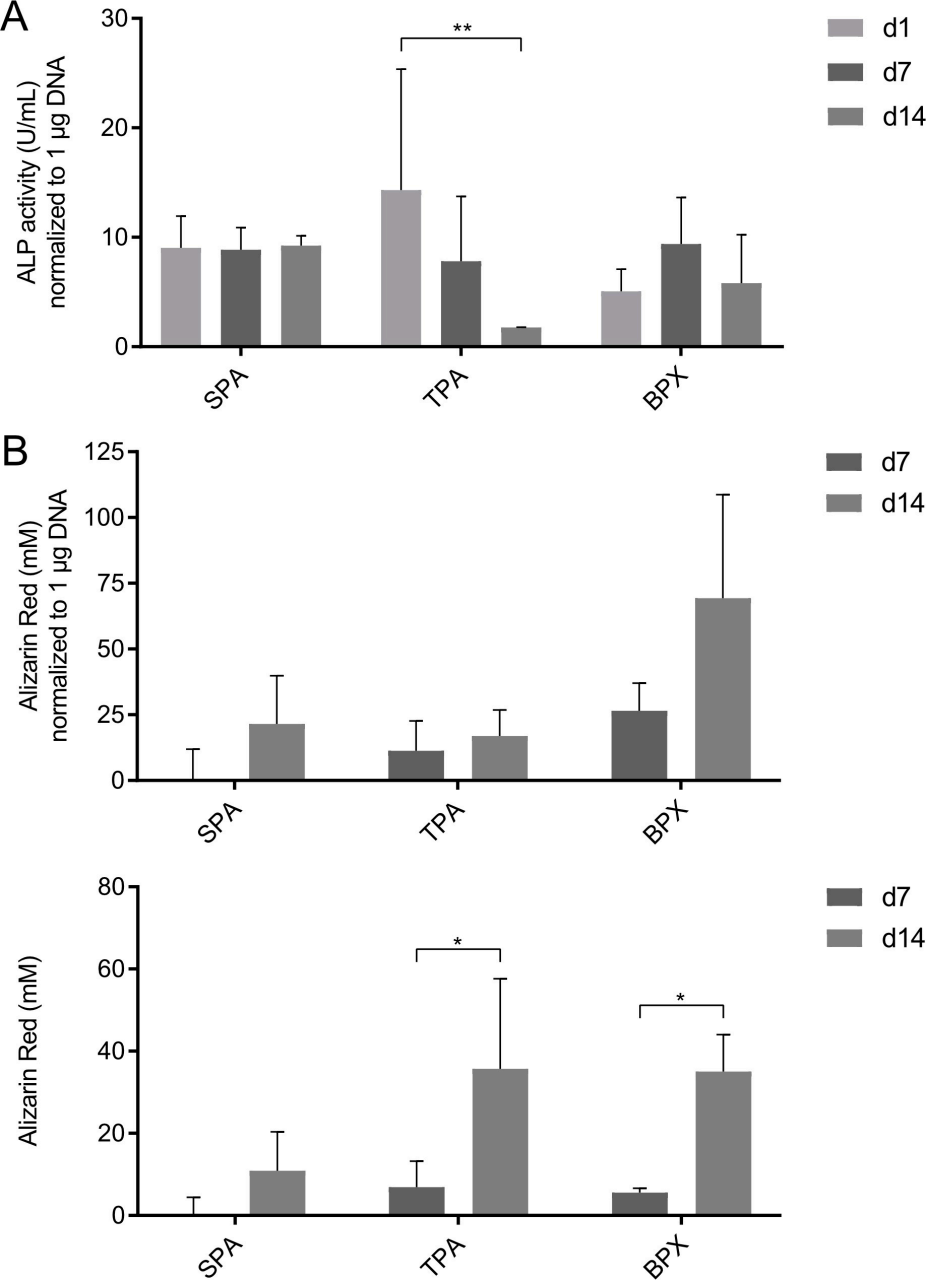
**ALP quantification (A) of cell-seeded constructs 1, 7 and 14 days and Alizarin Red S quantification (B) 7 and 14 days after seeding MSCs onto SPAs, TPAs and BPXs.** (A) ALP quantification was performed from supernatant retrieved from seeded grafts on day 1, 7 and 14. ALP levels were normalized to DNA amount (compare Fig 8). BPXs show the highest tentative, yet non-significant increase in ALP levels from day 1 to day 7. TPAs show a significant decrease from day 1 to day 14. (B) Alizarin Red S values were also normalized to corresponding DNA levels (Fig 9B, upper graph). Normalized data indicate the most prominent yet non-significant increase in calcification for BPXs. Alizarin Red S data not normalized to DNA however (Fig 9B, lower graph) indicate a significant increase in calcification for both BPXs and TPAs. Statistics are based on Tukey’s multiple comparison in conjunction with ANOVA (*n* = 3 MSC donors and 3 graft donors, two independent MSC-seeded constructs were quantified per donor, * *p* < 0.05, ** *p* < 0.01).

Further, ALP activity as an early marker for osteogenic differentiation was measured in order to assess MSC functionality on the grafts (Fig 9A). For this purpose, medium retrieved from MSC-seeded constructs was collected on day 1, 7 and day 14 and ALP levels were normalized to the corresponding DNA content (compare Fig 8) in order to cope with a potential impact of the cell numbers on the ALP activity. BPXs displayed a tentative, yet non-significant increase of normalized ALP activity from day 1 to day 7. ALP levels of SPAs showed no significant increase or decrease throughout the 14-day period while TPAs showed a significant decrease from day 1 to day 14 in the alkaline phosphatase activity as early osteogenic marker.

### Quantification of mineralization of cell seeded constructs by alizarin red S

Mineralization of MSC-seeded grafts was assessed by Alizarin Red S (Fig 9B) and included the subtraction of background values derived from grafts before cell seeding (see S1 Fig). Additionally, Alizarin Red S levels were normalized to the corresponding DNA content (Fig 9B, upper graph) to cope with a potential impact of the cell numbers on the degree of mineralization (compare Fig 8). After normalization to the DNA content BPXs displayed a tentative yet non-significant increase in mineralization from 26.6 mM/μg DNA on day 7 to 69.3 mM/μg DNA on day 14. However increased mineralization levels for both BPXs and TPAs were documented when alizarin quantification was not normalized to DNA content (Fig 9B, lower graph).

## Discussion

The increased incidence of bone defects, especially in cases of multifragmentary fractures or non-unions demand suitable bone grafts and therefore rising amounts of suitable allografts are required. Currently, several decellularization methods for allografts have been proposed but it still remains unclear which method results in favorable physiochemical properties or might be preferred in stem cell applications. Hence, the aim of this study was firstly to compare two decellularization methods concerning their decellularization capacity for bone grafts and secondly to investigate their impact on MSC functionality together with two commercially available grafts.

Based on histological examination, SEM examination and DNA quantification a higher efficacy in decellularization could be shown for SPAs in comparison to CPAs. This was documented by significantly higher amounts of DNA after decellularization in CPAs compared to SPAs, although CPAs were treated with DNAse in an additional step. Similarly, SEM images revealed marrow cavities filled with tissue underscoring incomplete decellularization in CPAs. SPAs on the other hand showed empty marrow cavities, revealing a rough surface structure and distinct trabecular structures. The increased clearance of marrow cavities in SPAs over CPAs might be due to the disrupting effect of sonication on cell membranes [40]. Additionally, mechanical energy exerted by sonication possibly helped to clear any residues [41]. In this study we treated SPAs as mentioned above by sonicating grafts at 20 kHz with an amplitude of 12 microns. This is in accordance to previous reports showing this frequency to have a cell membrane disrupting effect and a significant reduction in cell viability [40, 42]. It is important to note that the efficacy of decellularization for SPAs in our experiments was comparable to TPAs and BPXs as commercial standardized products.

Commercially available TPAs are generated by a combination of chemical and physical treatment steps including sonication, acetone treatment, osmotic treatment, sterilization via hydrogen peroxide solutions, serial dehydration and gamma irradiation [31, 43]. BPXs differ from all other grafts as they originate from a bovine source and are treated with heat (300°C), alkaline chemicals and sterilization using dry heart [43]. This process is supposed to remove any proteins or antigenic structures [44]. Nevertheless, assessment of protein content, or the presence of immune response mediating material was not in the scope of this present study.

However, initial biocompatibility testing based on extraction medium from the grafts showed a reduction in MSC viability for all grafts except TPAs. Results for TPAs are congruent with other published data showing favorable cell viability properties for TPAs [31, 45]. The reduction of cell viability in CPAs, SPAs and BPXs might be due to a variety of different reasons and its underlying biological mechanisms. The reduction in MSC viability for CPA extracts might be caused by chemical substances such as Triton X-100 and SDS, used in the decellularization process leaking out even after thorough washing. On the other hand many potential mediators such as TNF-alpha [46], cytochrome c [47] and miRNA released by cells during the decellularization method or in not completely decellularized materials might lead to reduced cell viability in MSCs treated with the extracts. Although the procedure includes intensive washings an impact of such molecules cannot be excluded but might be determined in future studies. BPX extracts also induced a reduction in MSC cell viability. As BPXs have been shown to be void of any cellular material (Fig 3) above mentioned explanations are not applicable to this graft. Though our findings are in line with other published data showing a reduction in cell viability [48, 49], to our knowledge no explanation has yet been given. Considering that BPXs mainly consists of hydroxyapatite and have a very porous consistency a tentative explanation could constitute the induction of apoptosis by nanoparticles [50]. Even though our results regarding BPXs are consistent with *in vitro* data it should be noted that a discrepancy between results from experiments *in vitro* and *in vivo* cannot be excluded. While *in vitro* testing often offers the ability to study cellular and molecular processes more closely, a direct translation to an *in vivo* setting might often be limited. In fact, BPXs have repeatedly been shown to integrate well *in vivo* by demonstrating large quantities of osteoid matrix depositions and being enveloped well by adjacent tissue [51, 52].

The impact of graft processing on the graft’s ability to host MSCs was assessed by examining cell morphology and cell density of seeded MSCs. CLSM revealed an elongated and spindle-like morphology of MSCs with a high cell density on SPAs and TPAs.

DNA quantification to monitor cell adhesion and proliferation on the different grafts in a quantitative manner indicated the best MSC growth and proliferation on TPAs. These data were further supported by the MTS data for the TPA extracts indicating a good biocompatibility and viability levels close to the controls, as well as by the morphological assessment as described above. Further DNA quantification demonstrated that the initial adhesion was similar for all tested constructs so that the good cell growth on TPAs might not be explained by differences in initial cell adhesion or technical issues associated with the seeding procedure.

In this context, EDX analysis of bone grafts indicated in TPAs the highest values for nitrogen and conversely the lowest Ca/N ratios, followed by SPAs. Human bone is composed of a mineral phase (hydroxyapatite), an organic phase (mainly collagen type I) and water [53]. A high At% of nitrogen, ubiquitous in organic compounds, might correlate with a high amount of collagen [54] in TPAs and SPAs. MSCs at the same time adhere readily to collagen type I [55]. This in turn could explain superior properties for MSC functionality in regard to TPAs but not for SPAs.

BPXs displayed the highest At% of calcium and phosphorous. Yet, Ca/P ratios were similar for all grafts and did not differ significantly. Shih et al. showed that calcium phosphate rich bone grafts can induce osteogenesis via a phosphate-adenosine signaling pathway [30]. It is interesting to note that while BPXs showed the highest At% of calcium and phosphorous they also demonstrated the highest tentative, yet non-significant increase in ALP activity. Additionally, quantification of mineralization by Alizarin Red S showed tentative (DNA normalized) respectively significant (not normalized) increase potentially associated with osteoinductive properties of calcium rich BPXs. Accordingly, BPXs showed high background values in Alizarin Red S assays. While this further substantiates the results from EDX spectroscopy, depicting BPXs as the grafts with the highest amount of Ca (Fig 4A), it also limits to some extend the interpretability of results on mineralization due to these high background levels despite of subtraction.

Biomineralization and hydroxylapatite deposition is well known to depend on ALP activity as it provides phosphates during these processes [56, 57] and further inactivates calcification inhibitors [58]. Furthermore, ALP is amongst the early markers of osteoblast differentiation which is highly prominent in the starting phase of the mineralization process [59, 60] but undergoes a downregulation when mineralization progresses. Accordingly, the significant decrease of ALP activity in TPAs along with the increase in mineralization (non-normalized data) reflects such a typical marker profile widely described for osteogenic differentiation of MSCs and further underlines the impact of TPAs on MSC functionality.

## Conclusions

The outcome of this study shows a higher efficacy in decellularization for SPAs over CPAs based on DNA quantification, histological and SEM evaluation. Moreover, the decellularization efficacy of SPAs was comparable to two commercial grafts (TPAs and BPXs) used as additional reference also in terms of commercially standardized products. Biocompatibility assessment based on extracts derived from decellularized grafts showed a decrease in MSC viability for SPAs and CPAs as well as for the commercially available BPXs. In contrast, biocompatibility was not impaired for TPAs which also showed a better performance after reseeding with MSCs as indicated by CLSM and DNA assessment in order to monitor cellular proliferation. Here, a significant increase in DNA throughout a two-week time frame could be shown. BPXs induced a tentative increase in ALP activity and mineralization in MSC potentially associated with the high calcium content. Even though SPAs extracts showed a noticeable reduction of in vitro biocompatibility, results after reseeding with MSCs were comparable to commercially available grafts used in this study. Nevertheless, in this present study TPAs combined the best *in vitro* biocompatibility and performance in terms of proliferation and osteogenic differentiation after reseeding with MSCs.

## Supporting information

S1 Fig Alizarin red S quantification of un-seeded SPAs, TPAs and BPXs as background. Background values of Alizarin Red S quantification were obtained by staining un-seeded grafts with Alizarin Red S solution and consecutively extracting and photocolorimetrically measuring the Alizarin Red S that attached to the un-seeded grafts. BPXs show the highest values, differing significantly to all other grafts. TPAs on the other hand display the lowest values. Statistics are based on Tukey’s multiple comparison in conjunction with ANOVA (*n* = 3, ** *p* < 0.01).
(TIF)
