# Supplementary figures and images for: Evaluation of bone allograft processing methods: Impact on decellularization efficacy, biocompatibility and mesenchymal stem cell functionality
Source: PLoS One. 2019 Jun 20;14(6):e0218404. doi: 10.1371/journal.pone.0218404 (PMC6586299; doi:10.1371/journal.pone.0218404)

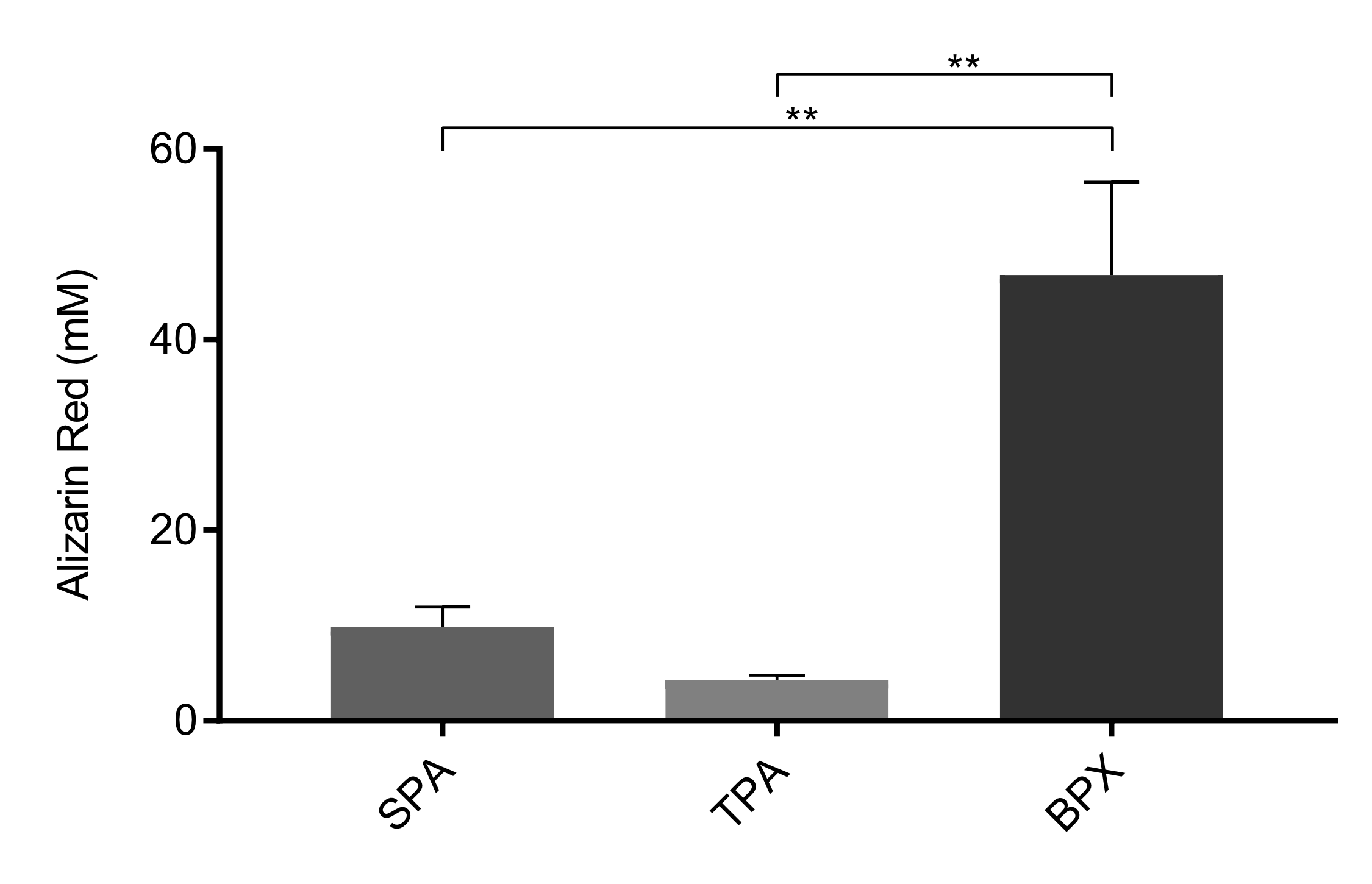

Supplement: S1 Fig — Background values of Alizarin Red S quantification were obtained by staining un-seeded grafts with Alizarin Red S solution and consecutively extracting and photocolorimetrically measuring the Alizarin Red S that attached to the un-seeded grafts. BPXs show the highest values, differing significantly to all other grafts. TPAs on the other hand display the lowest values. Statistics are based on Tukey’s multiple comparison in conjunction with ANOVA (n = 3, ** p < 0.01). (TIF) [file pone.0218404.s001.tif]
